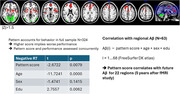# Regional amyloid shows associations with task‐related fMRI activation recorded 5 years earlier

**DOI:** 10.1002/alz.094698

**Published:** 2025-01-09

**Authors:** Christian G Habeck, Yaakov Stern

**Affiliations:** ^1^ Taub Institute for Research in Alzheimer’s Disease and the Aging Brain, Columbia University, New York, NY USA; ^2^ Columbia University, New York, NY USA; ^3^ Columbia University Irving Medical Center, New York, NY USA; ^4^ Cognitive Neuroscience Division, Columbia University, New York, NY USA

## Abstract

**Background:**

We were interested to test whether regional amyloid as measured with Florbetaben could be detected in fMRI activation patterns recorded 5 years prior.

**Method:**

Perceptual Speed was probed with 3 cognitive fMRI tasks (Digit Symbol Substitution, Letter Comparison, Pattern Comparison) for 324 participants, aged 20 to 80, at timepoint 1. A subsample of 63 participants (aged 51‐80) underwent Florbetaben amyloid scans 5 years later ( = timepoint 2), resulting in SUVR values in 68 regions of the FreeSurfer DK atlas. For these 63 participants, Principal Components analysis was conducted in the activation data that were averaged within participant across all 3 tasks. Correlations of the resulting pattern scores with regional amyloid were observed, adjusting for the covariates of age, sex and years of education. The relationship to task performance in the fMRI tasks ( = sign‐reversed RT) was observed too, again with the covariates age, sex, and years of education. All participants were healthy, without cognitive impairment.

**Result:**

The pattern score of the first principal component in the fMRI activation data in the subsample with N = 63 correlated with subsequent regional amyloid in 22 regions at p<0.05. In the larger sample of N = 324, the pattern score also correlated negatively with task performance, beyond the covariates of age, sex and years of education. The pattern consisted of positive loadings (Z>1.5) in frontal locations like anterior cingulate, superior and inferior frontal gyrus, bilateral insula, basal ganglia. The pattern showed negative loadings (Z←1.5) in occipital and cerebellar regions (see Figure).

**Conclusion:**

Regional amyloid had a robust correlation with an underlying fMRI activation pattern for Perceptual‐Speed tasks collected 5 years earlier. While in the subsample of 63 there was no relationship of the pattern with fMRI‐task performance, in a whole lifespan sample with N = 324, the amyloid‐associated activation pattern was *negatively* related to task performance. These findings suggest that amyloid can impact activation in a manner that is detrimental to task performance even in healthy older adults.